# Protrudin regulates FAK activation, endothelial cell migration and angiogenesis

**DOI:** 10.1007/s00018-022-04251-z

**Published:** 2022-04-04

**Authors:** Amita Arora, Annukka M. Kivelä, Ling Wang, Rimante Minkeviciene, Juuso H. Taskinen, Birong Zhang, Annika Koponen, Jing Sun, Michiko Shirane, You Zhou, Pirta Hotulainen, Camilla Raiborg, Vesa M. Olkkonen

**Affiliations:** 1 Minerva Foundation Institute for Medical Research, Biomedicum 2U, Tukholmankatu 8, 00290 Helsinki, Finland; 2 Centre for Cancer Cell Reprogramming, Faculty of Medicine, University of Oslo, Oslo, Norway; 3 Department of Molecular Cell Biology, Institute for Cancer Research, Oslo University Hospital, Oslo, Norway; 4 Systems Immunity Research Institute, Cardiff University School of Medicine, Cardiff University, Cardiff, UK; 5 Division of Infection and Immunity, Cardiff University School of Medicine, Cardiff University, Cardiff, UK; 6 Department of Molecular Biology, Graduate School of Pharmaceutical Sciences, Nagoya City University, Nagoya, Aichi Japan; 7 Department of Anatomy, Faculty of Medicine, University of Helsinki, Helsinki, Finland

**Keywords:** Endosomes, Focal adhesion kinase, mTOR, Vasculature, Zfyve27

## Abstract

During angiogenesis, endothelial cells form protrusive sprouts and migrate towards the angiogenic stimulus. In this study, we investigate the role of the endoplasmic reticulum (ER)-anchored protein, Protrudin, in endothelial cell protrusion, migration and angiogenesis. Our results demonstrate that Protrudin regulates angiogenic tube formation in primary endothelial cells, Human umbilical vein endothelial cells (HUVECs). Analysis of RNA sequencing data and its experimental validation revealed cell migration as a prominent cellular function affected in HUVECs subjected to Protrudin knockdown. Further, our results demonstrate that knockdown of Protrudin inhibits focal adhesion kinase (FAK) activation in HUVECs and human aortic endothelial cells (HAECs). This is associated with a loss of polarized phospho-FAK distribution upon Protrudin knockdown as compared to Protrudin expressing HUVECs. Reduction of Protrudin also results in a perinuclear accumulation of mTOR and a decrease in VEGF-mediated S6K activation. However, further experiments suggest that the observed inhibition of angiogenesis in Protrudin knockdown cells is not affected by mTOR disturbance. Therefore, our findings suggest that defects in FAK activation and its abnormal subcellular distribution upon Protrudin knockdown are associated with a detrimental effect on endothelial cell migration and angiogenesis. Furthermore, mice with global Protrudin deletion demonstrate reduced retinal vascular progression. To conclude, our results provide evidence for a novel key role of Protrudin in endothelial cell migration and angiogenesis.

**Supplementary Information:**

The online version contains supplementary material available at 10.1007/s00018-022-04251-z.

## Introduction

Endothelial cells (ECs) line the lumen of blood vessels and play a major role in the process of angiogenesis wherein new blood vessels are formed from pre-existing ones. Angiogenesis is triggered by a gradient of growth factors, primarily VEGF-A (hereafter referred as VEGF), in response to conditions such as hypoxia or lack of nutrient supply to tissue [1, 2]. The endothelial-derived tip cells form protrusive sprouts and start migrating towards the stimulus [3, 4]. EC migration is fundamental to the process of angiogenesis and involves membrane elongation to form polarized protrusions. This process is orchestrated primarily by integrin-mediated cell adhesion to the extracellular matrix and initiation of intracellular signal transduction, formation of focal adhesion complexes and cytoskeletal rearrangements that facilitate changes in cell shape essential for migration [5, 6].

The mechanistic target of rapamycin (mTOR) is an evolutionary conserved atypical serine/threonine kinase that exists in two complex forms in mammalian cells: mTORC1 and mTORC2. Both complexes have been extensively studied and shown to regulate cellular processes like cell proliferation, survival and motility [7]. p70S6K1 signaling activated by mTORC1 promotes phosphorylation of the focal adhesion proteins FAK and paxillin [8]. In addition, mTORC1 promotes cell motility by regulating the expression and activity of small GTPases (RhoA, CDC42 and Rac1) and thus F-actin reorganization [9]. Similarly, the mTORC2 effectors AKT and PKC promote cell migration by regulating Rho family GTPases [10, 11]. The study by Farhan et al. [12] suggested role of mTORC2 in sprouting angiogenesis in part by regulation of FAK activity and cytoskeletal remodeling. Emerging evidence proposes a key role of mTORC1 signaling in vascular endothelial functions and tumor angiogenesis, highlighting the need to further elucidate the regulation of this signaling pathway in endothelial cells [13, 14].

Protrudin/SPG33 is a multi-domain, endoplasmic reticulum (ER) transmembrane protein that functions in ER morphogenesis [15, 16] and at the contact sites of ER and endosomes [17]. The protein harbors a Rab11-binding domain (Rab11-BD) mediating interaction with recycling endosomes (RE), a low-complexity region (LCR) for association with Rab7 on late endosomes (LE), and a FYVE (Fab1-YOTB-Vac1-EEA1) domain. The FYVE domain interacts with endosomal PI3P or PI(4,5)P_2_ [17–20]. Initial studies focused primarily on the role of Protrudin in neurite outgrowth, wherein the kinesin-binding domain of Protrudin facilitates the loading of kinesin motor protein onto the endosomes. The loading of kinesin promotes the anterograde transport of endosomes, followed by their fusion with the plasma membrane (PM) and thus membrane elongation [17, 20–22]. Studies highlighting the cargo molecules transported anterogradely by Protrudin are currently emerging. Overexpression of Protrudin promotes axonal regeneration by facilitating transport of Rab11-positive recycling endosomes carrying molecules such as integrin α9 to the distal axons [23]. Moreover, Protrudin-mediated anterograde translocation promotes exocytosis of membrane type I matrix metalloproteinase (MT1-MMP) in breast cancer cells. Protrudin-mediated exocytosis is important for the invasiveness of these cancer cells [24]. A recent study by Hong et al. [25] demonstrated that Protrudin facilitates the translocation of lysosome-bound mTORC1 to the cell periphery and thereby its activation. However, the physiological relevance of Protrudin-mediated mTORC1 redistribution remains unclear.

Given that Protrudin promotes membrane outgrowth in neuronal and breast cancer cells, the protrusive behavior of ECs during sprouting angiogenesis prompted us here to investigate the putative role of Protrudin in ECs and angiogenesis per se.

## Methods

### Mice

The Protrudin/Zfyve27 global knockout C57Bl/6 J mouse strain B6-Cg-Zfyve27 < tm1Kei > , RBRC10084 [26], was generated by M. Shirane and obtained from the RIKEN BioResource Research Center (BRC; Koyadai Tsukuba-shi Ibaraki, Japan). Mice with a C57Bl/6 J-C57Bl/6NCrl background were maintained at the Laboratory Animal Center of the University of Helsinki at a temperature of 21–22 °C, 52% humidity and light cycle of 12∶12 h, on Global 16% rodent diet 2916 (Teklad, Harlan Laboratories, Indianapolis, IN). Litters for experiments were produced by mating of Zfyve27^±^ animals, and the progeny were genotyped by a PCR protocol provided by RIKEN BRC.

### Antibodies and reagents

The following antibodies (Ab) were used: anti-Protrudin (12680–1-AP, RRID:AB_10640298) from Proteintech Group (Rosemont, IL), anti-Lamp1(sc-20011, RRID:AB_626853) and anti-phospho-PERK (Thr981) (sc-32577, RRID:AB_2293243) from Santa Cruz Biotechnology (Dallas, TX); anti-phospho-FAK(Tyr397) (8556, RRID:AB_10891442), anti-mTOR (2983, RRID:AB_2105622), anti-Orai (3280, RRID:AB_2157441), anti-p70S6K (9202, RRID:AB_331676), anti-phospho-p70S6K(Thr389) (9205, RRID:AB_330944) from Cell Signaling Technology (Danvers, MA); anti-phospho-EIF2α/EIF2S1 (Ser52) (44-728G, RRID:AB_2533736) anti-FAK (AHO0502, RRID:AB_2536313) and anti-phospho-FAK(Tyr861) (44-626G, RRID:AB_2533703) from Thermo Fisher Scientific (Waltham, MA), anti-GAPDH (ab9485, RRID:AB_307275) from Abcam (Cambridge, UK), anti-Actin (A2066, RRID:AB_476693) was from Sigma-Aldrich (Saint Louis, MO). Anti-EEA1 (610,457, RRID:AB_397830), Mouse BD Fc Block™(anti-Mouse CD16/CD32 antibody, Clone 2.4G2, 553,141, RRID:AB_394656), PE-CF594 conjugated-CD31 (563,616, RRID:AB_2738320) from BD Horizon (San Jose, CA); PE/Cy7 conjugated-CD45 (103,113, RRID:AB_312978) was purchased from BioLegend (San Diego, CA). Cy5-, Alexa-488- and Alexa-568-conjugated secondary antibodies, Alexa-594-conjugated phalloidin, and Alexa-488-conjugated-Isolectin B4 were from Molecular Probes/Invitrogen (Carlsbad, CA). Puromycin was purchased from Gibco/Fisher Scientific (Loughborough, UK) and VEGF-A_165_ from R&D Systems (Minneapolis, MN), Hepatocyte Growth Factor (HGF) was obtained from Sigma-Aldrich (Saint Louis, MO).

### Cell culture

Human umbilical vein endothelial cells (HUVECs) and Endothelial Cell Medium 2 (ECM2) along with the supplement mix (ECGM2) were obtained from PromoCell (Heidelberg, DE). The supplement mix includes: Fetal Calf Serum (2% v/v), Epidermal Growth Factor (5 ng/ml), Basic Fibroblast Growth Factor (10 ng/ml), Insulin-like Growth Factor (R3 IGF-1) (20 ng/ml), Vascular Endothelial Growth Factor 165 (0.5 ng/ml), Ascorbic Acid (1 µg/ml), Heparin (22.5 µg/ml), and Hydrocortisone (0.2 µg/ml). Human Aortic Endothelial cells (HAEC) were procured from Lonza (Basel, CH). Cells were cultured in ECGM2 as described earlier [27]. Briefly, cells were seeded onto flasks/plates pre-coated with fibronectin 10 µg/ml–gelatin 0.05% (Sigma-Aldrich, Saint Louis, MO) and maintained in ECGM2 media at 37 °C in a humidified atmosphere with 5% CO_2_ until confluent. For VEGF stimulation, cells were starved in ECM2 containing 0.1% BSA (starvation media) for 5 h prior to lysis and then stimulated with or without 50 ng/ml VEGF (R& D systems) for time periods as indicated.

MDA-MB-231 cells were maintained in RPMI Sigma-Aldrich (Saint Louis, MO) supplemented with 10% FCS, 2 mM glutamine (Thermo Fisher Scientific, Waltham, MA), 100 U/ml penicillin, and 100 μg/ml streptomycin at 37 °C with 5% CO_2_. CRISPR/Cas9-mediated deletion of ZFYVE27 (Protrudin) in MDA-MB-231 cells was described in [24].

### cDNA constructs and production of recombinant lentiviruses

Human wild-type (WT) Protrudin (in pEGFP-C), LCR-domain deficient (Protrudin ΔLCR) and kinesin-binding domain-deficient (Protrudin ΔKIF) constructs have been described earlier [17]. GFP-tagged Protrudin ΔKIF was generated by transferring the cDNA from pcDNA3-myc into EcoRI/ApaI restriction sites of the pEGFP-C vector (Clontech/Takara Bio, Mountain View, CA). A double deletion mutant Protrudin ΔLCRΔFYVE was generated by deleting by PCR the carboxy-terminal FYVE domain of the pEGFP ΔLCR construct. For Gateway cloning, GFP was removed from the wild-type and mutant constructs and the cDNA cloned into the entry vector pENTR2B (Invitrogen/Thermo Fisher, Carlsbad, CA). The obtained constructs were recombined into the Gateway destination plasmid pLenti6.3/V5-DEST by the Helsinki University Genome Biology Unit (GBU, Helsinki Institute of Life Science HiLIFE and Biocenter Finland); a lentivirus produced from empty pENTR2B recombined with pLenti6.3/V5-DEST was used as a negative control and referred as Mock in “Results”. Protrudin-WT sequence in pLenti6.3/V5-DEST was made shRNA resistant by introducing three silent mutations by site-directed mutagenesis. For Protrudin knockdown MISSION® shRNAs TRCN0000138667 (shProtrudin#1) and TRCN0000134203 (shProtrudin#2) in the pLKO.1 lentiviral vector, TRCN0000039772 for Raptor knockdown (shRaptor) and control shRNA SHC002 (shNT) in the same vector were purchased from Sigma-Aldrich. Unless specifically mentioned, shProtrudin#1 was used for experiments.

### Lentivirus production and transduction

Lentiviruses were packaged by the Biomedicum-Helsinki Functional Genomics Unit (FUGU, HiLIFE and Biocenter Finland) and used either as fresh packaging cell culture supernatants or as concentrated stocks stored at − 80 °C (for in vitro angiogenesis assay) with p24 titers of 2 × 10^7^ − 2 × 10^8^ pg/ml. For knockdown, cells were incubated for 48 h with the lentiviruses at a multiplicity of infection (MOI) of 10, followed by 16-h antibiotic selection with 2.5 µg/ml puromycin (Gibco/Thermo Fisher Scientific, Waltham, MA). For overexpression, cells were incubated for 48 h with the lentiviruses at a multiplicity of infection (MOI) of 20 for Mock and Protrudin-WT, MOI 10 for Protrudin ΔLCRΔFYVE, and MOI 5 for Protrudin ΔKIF to ensure a similar level of overexpression for all constructs.

### Plasmid transfection

Plasmid transfections of HUVECs were done by electroporation using the Nucleofector™ Kit (Lonza, Basel, CH) according to Amaxa™ Optimized Protocol. Cells were transfected with 0.9 µg of plasmid DNA and plated onto 13 mm coverslips pre-coated with fibronectin 10 µg/ml–gelatin 0.05% in a 24-well plate containing ECGM2 media. The cells were incubated for 24 h prior to staining.

### Tube formation assay

In vitro angiogenesis assay kit was purchased from Millipore (Burlington, MA) and tube formation was assayed as per manufacturer’s instructions. Briefly, ECMatrix™ was plated on 96-well plates and incubated for 1 h at 37 °C to allow polymerization. At 48 h post-lentiviral transduction of HUVECs, the cells were seeded on top of the ECMatrix™ and incubated for 5 h. Endothelial cells, when seeded and incubated on the ECMatrix™, form capillary-like structures known as tubes. The neighboring tubes then fuse to form a mesh/loops. Images of the tubes and loops were taken with a 4 × objective of Invitrogen™ EVOS™ microscope. The total number of tubes and loops, and the length of tubes were analyzed with the Wimasis (Córdoba, Spain) automated image analysis platform.

### TUNEL and Annexin V-based apoptosis/necrosis assay

TUNEL assay kit-FITC was purchased from Abcam and the assay was performed according to the manufacturer’s instructions. Briefly, post-lentiviral transduction and puromycin selection, shNT and shProtrudin HUVECs were trypsinized, washed and fixed with 1% paraformaldehyde. Cells were then transferred to ice-cold 70% ethanol, washed, and incubated with staining solution containing FITC-labeled dUTP, followed by incubation in propidium iodide solution. Cells were analyzed with BD Accuri C6 Flow Cytometer (BD Biosciences, San Jose, CA).

RealTime-Glo™ Annexin V Apoptosis and Necrosis Assay kit was purchased from Promega (Madison, WI) and the assay was carried out as per manufacturer’s instructions. Briefly, 20 × 10^3^ cells were seeded in a 96-well plate along with the lentiviral construct as indicated in the *Results* section. On the day of assay, media was removed and warm ECGM2 media containing luminescent Annexin V substrate and fluorescent necrosis detection reagent was added to the cells, followed by luminescence and fluorescence measurements using an EnSpire multimode plate reader (Perkin Elmer, Waltham, MA).

### Western blotting

Cells were lysed in 0.5% NP-40, 5% glycerol, 150 mM NaCl, 50 mM Tris–HCl pH 8.0, 5 mM MgCl_2_ with complete Protease Inhibitor and phosphatase inhibitor Cocktail (Roche) and cleared by centrifugation at 13,000xg at 4 °C. Proteins were separated by SDS–PAGE and blotted onto PVDF membrane (BioRad). Immunodetection was performed using HRP-labeled secondary antibodies and enhanced chemiluminescence (ECL; Thermo Scientific) and a BioRad ChemiDoc™ Touch imaging system. The bands were quantified using ImageJ/Fiji.

### Next-generation RNA sequencing

HUVECs were transduced with shRNA against Protrudin (shProtrudin#1) or shNT lentivirus (MOI 10) (*n* = 4) for 48 h, followed by 16-h antibiotic selection with 2.5 µg/ml of puromycin. The RNA was isolated with the Qiagen (Hilden, DE) RNeasy™ Mini kit according to the manufacturer’s protocol. NEBNext Ultra Directional RNA Library Prep Kit was used to generate cDNA libraries for sequencing. Sequencing was performed with Illumina NextSeq system at the Functional Genomics Unit, University of Helsinki (the Helsinki Institute of Life Science, HiLIFE and Biocenter Finland). Analysis of the sequences was performed using Chipster suite [28] using the following workflow: FASTQ reads were trimmed using Trimmomatic [29], trimmed pair-ended reads were aligned to the Homo_sapiens.GRCh38.95 genome using STAR [30], aligned reads were counted using HTSeq [31], differential expression analysis was performed using DESeq2 [32] with a Benjamini–Hochberg (BH) adjusted P value cutoff of 0.05 and Ensembl identifiers were annotated using BioMaRt [33]. Differentially expressed genes between Protrudin knockdown and control cells (log2-fold change <  = -1 or >  = 1; adjusted *p*-value < 0.05) were selected for analysis by the Ingenuity Pathways Analysis tool (IPA; Qiagen, Hilden, Germany). Enrichment of the genes in the pathways was assessed in comparison with a reference set in the whole Ingenuity pathway knowledge base [34]. Volcano plot was generated using GraphPad Prism.

### Quantitative real-time PCR

RNA was isolated from HUVECs with Purelink® RNA Mini Kit (Thermo Fisher Scientific, Waltham, MA), according to the manufacturer's instructions. cDNA was synthesized with SuperScript® VILO™ synthesis Kit (Invitrogen, Carlsbad, CA). Quantitative RT-PCR was done using Roche SYBR-Green® master mix and a LightCycler 480 II Real-Time PCR system (Roche Applied Science, Penzberg, Germany). Crossing point (Cp) value was calculated and normalized to Cp values of housekeeping genes succinate dehydrogenase complex subunit A (SDHA) and beta-actin.

### Immunofluorescence staining and microscopy

Cells were grown on coverslips pre-coated with gelatin/fibronectin. For immunostaining, cells were fixed and permeabilized in PBS/0.1% Triton X-100 for 5 min followed by blocking in PBS/5% bovine serum albumin (BSA). The cells were then stained using the indicated primary antibodies for 1 h, washed three times with PBS, stained with secondary antibodies for 1 h, and washed three times in PBS. The cells were mounted in Mowiol containing 5 μg/ml DAPI (Life Technologies). The following antibodies were used anti-Lamp1 (Santa Cruz), anti-phospho-FAK (Tyr861) (Life Technologies), anti-mTOR (Cell Signaling Technology). Images were acquired using a Leica SP8X confocal microscope equipped with a 63 × oil objective and then deconvolved using the Huygens essential software. Deconvolved projections were analyzed using Fiji. For quantification of protrusions, cell processes with length greater than the longest diameter of the nucleus were counted, and the percentage of these cells among the total number of cells overexpressing Protrudin Wt or ΔKIF (or EGFP as a mock control) was determined. Quantification of perinuclear distribution of Lamp1 or mTOR endosomes was done using Fiji. Dots or clusters of Lamp1 or mTOR were segmented using manual threshold to measure the total fluorescence intensity in a cell. Intensity of such dots in the perinuclear region (defined by a fixed region around the DAPI-positive nuclei) was calculated from the total intensity of the segmented dots in the defined region. Data was expressed as (total fluorescence intensity in the perinuclear region/total fluorescence intensity in the whole cell) × 100. For calculation of stress fiber-positive cells, maximum projection of images with comparable focus plane for shNT and shProtrudin samples were used to score cells with stress fibers. Stress fibers were identified as actin filaments extending across the longitudinal cell axis. For each image, the percentage was calculated as number of stress fiber positive cells/total number of cells (DAPI-positive cell nuclei) × 100.

### xCELLigence real-time cell migration assay

Migration of HUVECs transduced with shNT or shProtrudin lentiviruses was measured using an xCELLigence RTCA DP instrument (ACEA Biosciences) equipped with CIM-plate 16 [27]. Prior to the xCELLigence measurements, cells were starved for 2 h in ECM2 media containing 0.1% BSA. After the starvation period cells were trypsinized and seeded at a density of 3 × 10^4^ cells/well into upper chamber of CIM-plate 16 containing starvation media. The wells of the lower chamber were loaded with ECGM2 media with double supplements and 50 ng/mL VEGF. As the cells migrated towards the chemoattractant across microelectronics sensors integrated at the bottom side of upper chamber, the impedance was measured for 18 h at 15-min intervals. The measurements were recorded and analyzed using Real-Time Cell Analysis (RTCA) Software 1.2 (ACEA Biosciences, San Diego, CA).

### Wound closure assay

Migration of HUVECs was assessed using IncuCyte™ ZOOM Scratch Wound assay (Essen BioScience, Hertfordshire, UK) as described earlier [27]. 3 × 10^4^ HUVECs were seeded in a 96-well Image Lock plate along with shNT or shProtrudin lentivirus in ECGM2 media. Cells were incubated for 48 h at 37 °C in a humidified atmosphere with 5% CO_2_, followed by puromycin selection for 16 h. Post-selection, cell monolayer was washed once with PBS and a uniform scratch wound was made in each well using IncuCyte Wound maker. Floating cells were removed by a PBS wash and fresh ECGM2 complete media was added. Cell migration was imaged every 2 h for a total of 24 h using IncuCyte Zoom microscope. Wound closure was quantified as % relative wound density using IncuCyte Cell migration software. The Relative Wound Density (RWD) metric of IncuCyte’s integrated software is self-normalizing for changes in cell density which may occur outside the wound as a result of cell proliferation.

### Subcellular fractionation

The protocol as described earlier [35] was followed with minor modifications. Briefly, HUVECs were collected in hypotonic lysis buffer (10 mM HEPES- KOH pH 7.2, 0.25 M sucrose, 1 mM EDTA, 1 mM MgOAc) containing protease and phosphatase inhibitors on ice. Cell membranes were disrupted by forcing them 100 × through a 23-gauge needle and nuclei were removed by centrifugation at 1,000xg for 10 min. The plasma membrane fraction was collected by centrifugation at 10,000xg for 10 min and endosomal/cytoplasmic fractions at 100,000xg centrifugation for 1 h. All fractionation steps were performed at + 4 ◦C. Fractions were dissolved in Laemmli sample buffer for immunoblotting.

### Isolation of endothelial cells from murine lungs and cerebrum

Mice lung endothelial cells were isolated as described by Xu et al. [36], with minor modifications. Briefly, lungs were minced, digested with 0.3% collagenase type IV (Invitrogen), and sieved through a 70 μm pore cell strainer (BD Falcon, Atlanta, GA). Cells were then centrifuged for 5 min, 500 rpm at 4 °C. 1 × 10^6^ cells were incubated with 1:100 dilution of Fc Block (BD Pharmingen, San Diego, CA) added to the cells for 30 min at 4 °C. Cells were then stained with PE/Cy7 conjugated anti-CD45 and PE-CF594 conjugated anti-CD31 for 1 h at 4 °C. Post-staining, cells were washed with FACS buffer (PBS supplemented with 0.5% BSA). CD45^–^CD31^+^ ECs were sorted with BD Influx Cell Sorter (BD Biosciences).

For isolation of ECs from mice cerebrum, cells were isolated using the Neural Tissue Dissociation Kit (P) and Myelin Removal Beads II (Miltenyi Biotec, Bergisch Gladbach, GE), as described previously [37, 38]. Cells were first blocked with Mouse BD Fc Block (clone 2.4G2, BD Pharmingen) on ice for 30 min and then stained for 1 h at 4 °C with gentle rotation with a combination of fluorophore-conjugated anti-mouse Abs as described above for isolation of ECs from mice lung tissue.

### Fluorescence microscopy analysis of mouse retina

Eyes were removed from P7 mice and fixed with 4% paraformaldehyde/PBS for 2 h on ice [39]. Retinas were dissected and blocked in 1% bovine serum albumin, 0.3% Triton X-100 in PBS overnight at 4 °C. They were then washed three times for 10 min with PBS and incubated overnight at 4 °C with Alexa 488 conjugated-Isolectin B4 (1:100). The retinas were washed twice for 15 min in PBS, 0.3% Triton X-100 and twice for 10 min in PBS, re-fixed in 4% paraformaldehyde for 10 min at room temperature and washed once in PBS/0.1% Triton X-100 and then in PBS, before flat-mounting on glass microscope slides using Mowiol (Southern Biotech, Birmingham, AL). Confocal microscopy was carried out on a Leica (Wetzlar, DE) TCS SP8 X confocal microscope. Retinal vascular progression was quantified by measuring the distance of vessel growth from the optic nerve to the periphery.

### Statistics

Statistical analysis was done using GraphPad Prism version 7.0e. Data was tested for normalization using Shapiro–Wilk normality test. For pairwise comparisons of two treatment groups, Student’s t-test was used. An unpaired two-tailed t-test was used to test two samples with equal variance, and a one-sample t-test was used in the cases where the value of the control sample was set to 1. For more than two samples, one-way Anova was used with suitable multiple comparison test. *p*-value of less than 0.05 was considered statistically significant. Experimental group sizes are indicated in the figure legends.

## Results

### Protrudin regulates angiogenic tube formation in vitro

**Fig. 1:**
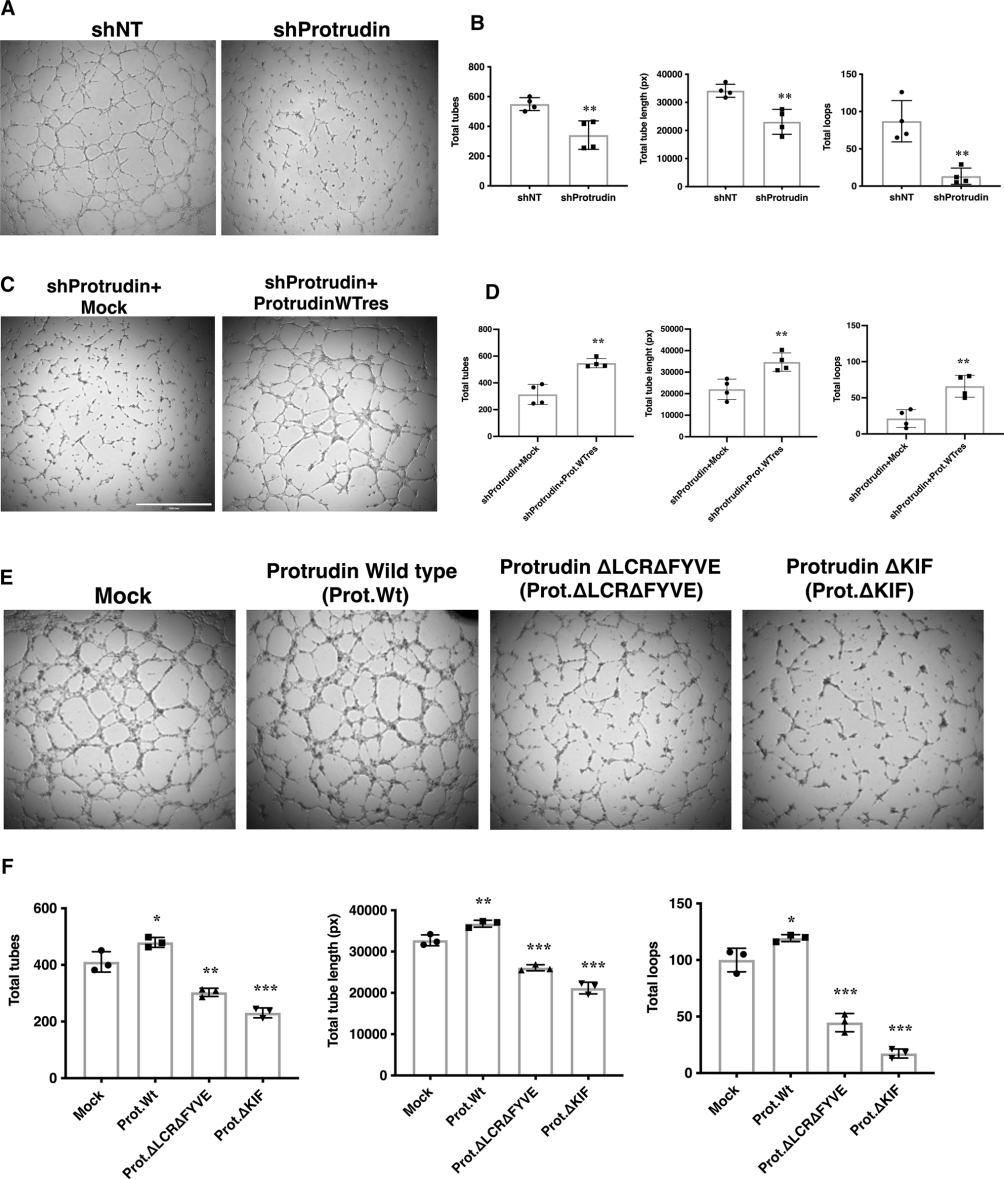
Protrudin regulates angiogenic tube formation in vitro. **a** HUVECs were transduced for 48 h with shRNA lentiviruses targeting Protrudin (shProtrudin) or a non-targeting shRNA (shNT). Cells were seeded in triplicate on the Millipore matrix in a 96-well plate containing ECGM2 complete media and incubated for 5 h. Phase contrast images were then taken of the tubes formed, 4 fields from each well. The experiment was done four times, a total of at least 40 fields per condition were imaged. Representative images are shown. **b** Bar diagram demonstrating quantification of the number of tubes, the length of tubes, and the number of loops in control versus Protrudin knockdown cells. Data represent mean ± SD of four independent experiments, two-tailed Student’s *t*-test.^**^p < 0.01. **c** HUVECs were transduced with shProtrudin + Mock lentivirus or shProtrudin + Protrudin-wild-type rescue lentivirus (shProtrudin + Prot.WTres) and then seeded on a Millipore matrix for tube formation assay as described in (**a**). **d** Bar diagram demonstrating quantification of the number of tubes, the length of tubes, and the number of loops in shProtrudin + Mock versus shProtrudin + Prot.WTres cells. Data represent mean ± SD of four independent experiments, two-tailed Student’s *t*-test. ^**^p < 0.01. **e** HUVECs were transduced with either empty (Mock) or Protrudin (wild-type or domain-deleted) lentiviral constructs for 48 h. Cells were then trypsinized and seeded on the Millipore matrix as described in (**a**). Experiment was done thrice with wells seeded in triplicate per sample each time; representative images are shown. Scale bar = 1000 μm. **f** Bar diagram demonstrating quantification of the number of tubes, the length of tubes, and the number of loops in control vs. Protrudin wild-type or domain-deleted protein expressing samples as indicated. Data represent mean ± SD of three independent experiments. One-way ANOVA followed by Dunnett’s multiple comparisons test ^*^p < 0.05, ^**^p < 0.01, ^***^p < 0.001 as compared to Mock

Angiogenesis involves sprouting of endothelial cells that begin to migrate toward a stimulus. The migratory sprouts eventually fuse to form vascular tubes that develop into mature vessels harboring a functional lumen [40, 41]. To investigate the role of endogenous Protrudin in this process in vitro, we carried out tube formation assays. For this, HUVECs were transduced with a lentivirus expressing shRNA that targets Protrudin (shProtrudin#1, hereafter mentioned as shProtrudin) or a non-targeting control (shNT). shProtrudin effectively reduced expression of the endogenous protein by nearly 70% as confirmed by western blot analysis (Suppl. Fig. S1a,  < 0.001). Cells were then seeded on ECMatrix™ supplemented with growth factors and imaged after 5 h. Silencing of Protrudin impaired the ability of HUVECs to form angiogenic tubes with significant reductions in the number of tubes (by 45%), tube length (by 42%), and the number of loops (by 78%) as compared to control cells transduced with shNT lentivirus (Fig. 1a, b). Importantly, the angiogenic tube formation defects could be rescued in shProtrudin HUVECs by overexpression of shRNA-resistant wild-type Protrudin (Prot.WTRes.) (Fig. 1c, d). As previously reported [25], knockdown of Protrudin does not induce ER stress in HUVECs as the knockdown had no effect on the phosphorylation level of PERK and eIF2α as compared to shNT cells (Suppl. Fig. S2a). To test if the reduction in tube formation was due to adverse effect of Protrudin knockdown on cell survival, we performed TUNEL apoptosis assay. For this, cells were fixed in 1% PFA, labeled with dUTP-FITC and analyzed by flow cytometry. As shown in Suppl. Fig. S2b, there was no significant difference in dUTP labeling between shProtrudin vs. shNT samples, suggesting that Protrudin knockdown does not induce apoptosis in HUVECs. To further verify that cell viability is not compromised, we performed Annexin V-based apoptosis and necrosis assay. The results confirmed that the knockdown of Protrudin did not induce cell death in HUVECs (Suppl. Fig. S2c).

As previously reported, Protrudin is a multi-domain protein with each domain playing a crucial role in cell membrane elongation, protrusion formation, and anterograde translocation of endosomes [17]. To confirm if the endosome translocation by Protrudin affects angiogenic tube formation, we overexpressed wild-type or domain-deleted constructs of the protein and assayed the in vitro tube formation by HUVECs. The level of overexpression was confirmed by western blot (Suppl. Fig. S1c); Cells overexpressing Protrudin were seeded on ECMatrix™ and imaged after 5 h. Overexpression of wild-type Protrudin (Protrudin-WT) significantly increased the length and number of tubes and the number of loops as compared to Mock transduced cells (Fig. 1e, f). Overexpression of Protrudin construct with double deletion of endosome-binding FYVE and LCR domains significantly impaired the tube forming ability of HUVECs as compared to controls (Fig. 1e, f). The construct with the KIF-binding domain deleted (ΔKIF) showed an even more drastic inhibitory effect, with a decrease in the number of tubes by 44%, tube length by 35%, and the number of loops by 83% as compared to controls (Fig. 1e, f). Overexpression of Protrudin-WT or domain-deleted constructs did not affect HUVEC survival as determined by Annexin V-based apoptosis and necrosis assay (Suppl. Fig. S2d).

**Fig. 2:**
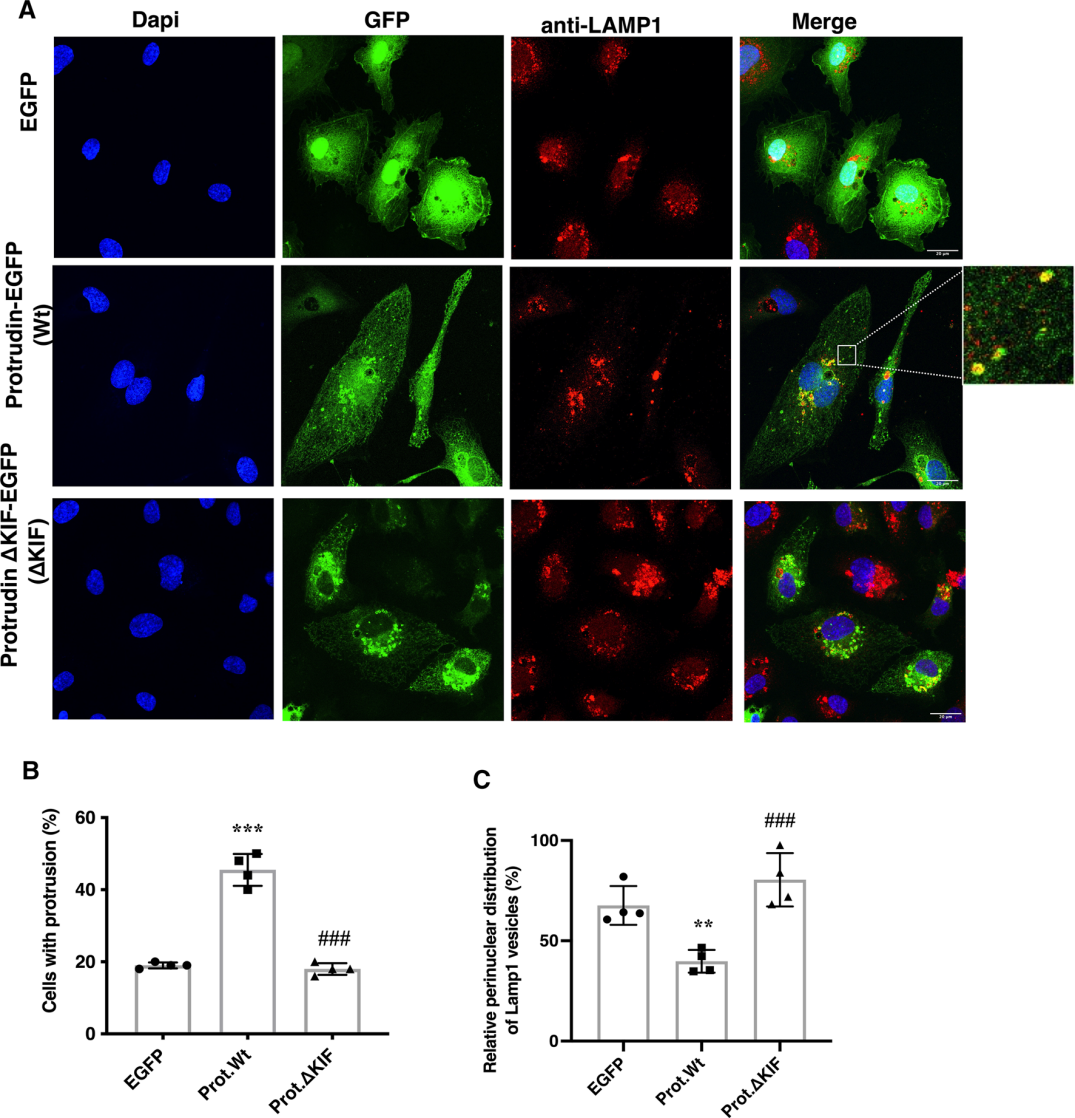
Protrudin regulates late endosome/lysosome (LE/Lys) distribution and protrusion formation in endothelial cells. **a** Deconvolved confocal microscopic images of HUVECs expressing electroporated plasmid constructs encoding GFP (control), Protrudin-GFP (Protrudin-WT) or KIF-binding domain-deleted Protrudin-GFP (Protrudin-ΔKIF). After 24 h of transfection in ECGM2 complete media, cells were fixed in 4% PFA and immunostained with anti-Lamp1 antibody. The insert highlights dispersed Lamp1 vesicles colocalized with Protrudin-WT representing ER-endosome contact sites. Scale bar = 20 μm. **b** Quantification of protrusion formation in HUVECs transfected as in (**a**). Data represent mean ± SD of four independent experiments (200 cells per condition), one-way ANOVA followed by Tukey’s multiple comparisons test. ^***^p < 0.001 as compared to EGFP transfected control, ^###^*p* < 0.001 as compared to Protrudin-WT. **c** Quantification of perinuclear distributed Lamp1-positive LE/Lys in HUVECs transfected as in (**a**). Bars represent the percentage of perinuclear Lamp1 vesicles from total Lamp1-positive vesicles in a cell. Data represent mean ± SD of four independent experiments. One-way ANOVA followed by Tukey’s multiple comparisons test ^**^p < 0.01 as compared to EGFP transfected sample, ^###^*p* < 0.001 as compared to Protrudin-WT

Angiogenic tube formation involves the extension of plasma membrane protrusions. Therefore, to study if Protrudin affects membrane extension in HUVECs, cells were transfected with EGFP-tagged Protrudin-WT or ΔKIF constructs. Confocal microscopy analysis showed a significant increase in the number of protrusions upon Protrudin-WT overexpression (Fig. 2a, b). However, the construct with the KIF-binding domain deleted failed to show this effect. Also, in line with the previous study [17], while overexpression of Protrudin-WT dispersed endosomes toward the cell periphery, expression of the KIF-binding domain-deleted construct allowed perinuclear clustering of late endosomes/lysosomes (LE/Lys) similar to the EGFP control (Fig. 2c).

To conclude, the above data demonstrate that Protrudin plays an important role in EC tube formation. Both the endosome-binding and the kinesin-binding domains of Protrudin are important for this function, suggesting that it involves the previously documented role of Protrudin in endosome translocation.

### RNAseq of HUVECs subjected to Protrudin knockdown

**Fig. 3:**
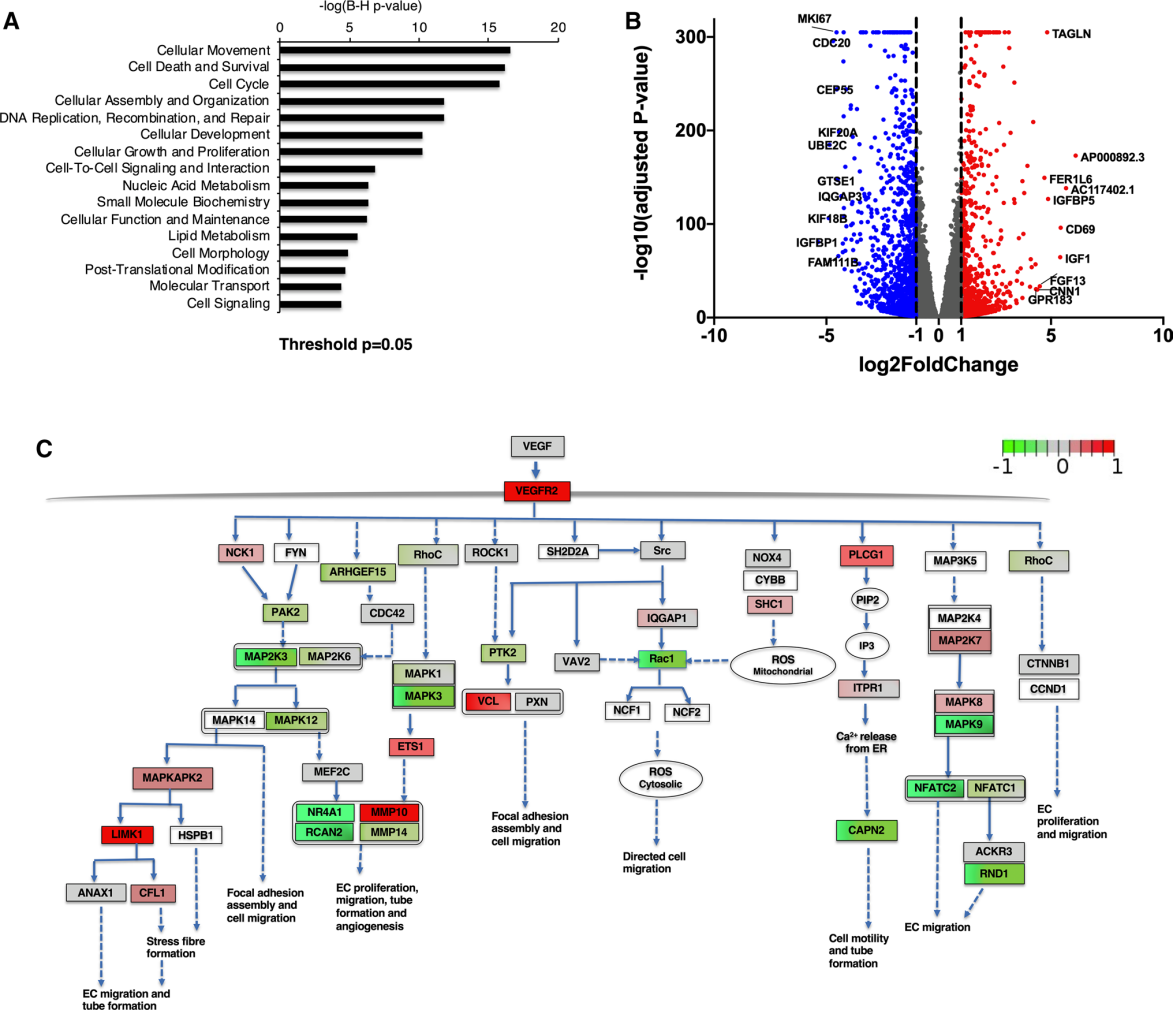
Cellular and molecular functions affected by Protrudin knockdown in HUVECs. **a** The molecular and cellular functions (RNA sequencing analysis) significantly affected in the shProtrudin cells were identified. *P* < 0.05 after multiple test adjustment was considered statistically significant (**b**) Volcano plot demonstrating differentially expressed genes between Protrudin knockdown and control HUVECs (adjusted *p* < 0.05). Significantly altered mRNAs with > twofold down- or upregulation are indicated in blue and red color, respectively. Top 10 up- and downregulated genes are identified. **c** VEGF-VEGFR2 signaling network to promote cell migration in endothelial cells illustrated using the Wikipathways database. Red boxes mark upregulated and green boxes downregulated genes upon Protrudin knockdown based on the indicated log2-fold change scale; adjusted *p*-value < 0.05. The brighter the color the higher is the log2-fold change value; uncolored box denotes not significant. *Note:* Many interconnections within the network have not been indicated here, to keep the network presentation simple. The main purpose of this network is to indicate the genes involved in endothelial cell migration that are significantly altered upon Protrudin knockdown in HUVECs

To further understand the function of Protrudin in endothelial cells, we performed next-generation RNA sequencing of HUVECs lentivirally transduced either with shRNA against Protrudin or non-targeting control shRNA (shNT, control). The analysis of significantly altered genes revealed ‘Cellular movement’, ‘Cell death and survival’, ‘Cell cycle’ and pathways involving Cellular assembly, organization and morphology as well as ‘Molecular transport’ among the major pathways significantly affected by the Protrudin knockdown (Fig. 3a). The distribution of the transcriptome data is displayed as a volcano plot in Fig. 3b and the 10 most up- and downregulated mRNAs (based on fold change) are identified in the plot. Of note, these mRNAs included IQGAP3, FGF13, TAGLN encoding proteins involved in cytoskeletal signaling and cell migration [42–45]. The entire transcriptome data are included in Suppl. Table S1.

EC migration is obligatory to the process of angiogenic tube formation. Therefore, we next analyzed the mRNA alterations in the VEGF-VEGFR2 signaling-mediated EC migration pathway upon Protrudin knockdown. Figure 3c demonstrates gene expression alterations for some of the key molecules downstream of VEGF-triggered VEGFR2 phosphorylation that regulate EC migration. VEGF-VEGFR2 signaling network as indicated in Wikipathways was used as a reference to plot this signaling network (https://www.wikipathways.org/index.php/Pathway:WP3888) [46, 47].

### Protrudin promotes endothelial cell migration

**Fig. 4:**
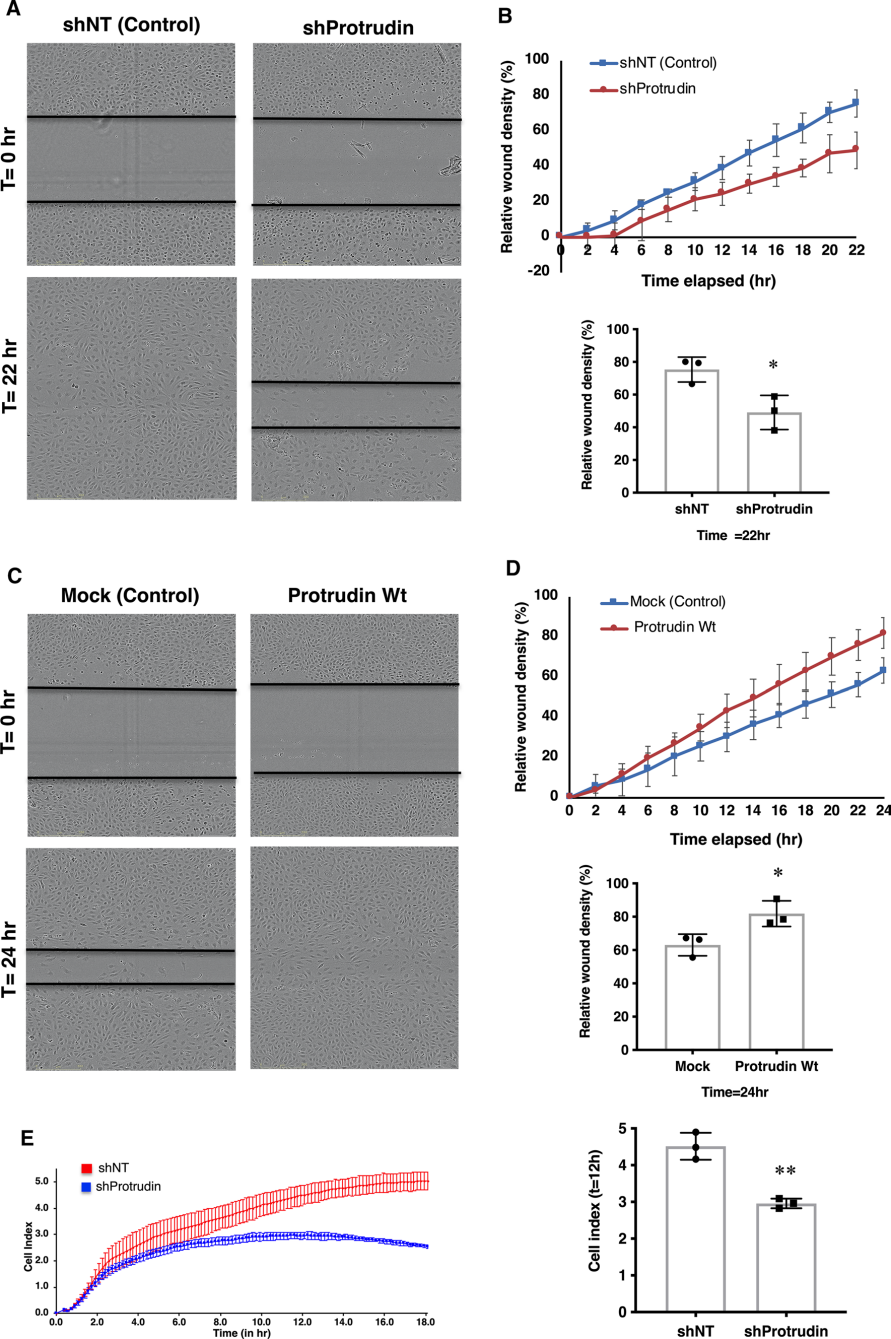
Protrudin promotes endothelial cell migration. **a** Representative images of wound closure in HUVECs transduced with Protrudin knockdown or non-targeting lentivirus in ECGM2 complete media, at t = 0 and t = 22 h. Scale bar = 300 μm. **b** Line diagram representing cell migration profile of HUVECs towards the wounded area over a period of 22 h. The bar graph represents relative wound density (%) in control (shNT) vs. Protrudin knockdown HUVECs for three independent experiments; two-tailed Student’s t-test, ^*^p < 0.05. Data represent mean ± SD. **c** Wound-healing assay using HUVECs overexpressing Protrudin wild-type (Protrudin-WT) or Mock (Control) lentiviral construct. At 48 h post-transduction, a wound was made in confluent monolayers and cells were imaged for 24 h. Representative images at *t* = 0 and *t* = 24 h are shown. Scale bar = 300 μm. **d** Line diagram representing cell migration profile of HUVECs towards the wounded area over a period of 24 h. The bar graph represents relative wound density (%) in Mock vs. Protrudin overexpressing HUVECs. Data represent mean ± SD, ^*^p < 0.05. **e** Migration profile of shProtrudin HUVECs compared to shNT (non-targeting shRNA) cells measured in real-time using an impedance-based migration assay on xCELLigence RCTA DP instrument. Bar graph representing migration cell index in Control (shNT) vs. Protrudin knockdown HUVECs at 12 h. Experiment was done thrice. Data represent mean ± SD, two-tailed Student’s *t*-test ^**^p < 0.01

Analysis of the RNAseq data indicated cell movement as the cellular function most severely affected upon Protrudin knockdown in HUVECs. To validate the role of Protrudin in EC migration, we first performed two-dimensional scratch wound assays. HUVECs were seeded on plates pre-coated with fibronectin/gelatin along with Protrudin or control shRNA lentivirus for 48 h, followed by puromycin selection. Post-selection, a uniform scratch wound was made using Incucyte™ wound maker and cell migration was observed for 22 h (Fig. 4a). Knockdown of Protrudin retarded the migration profile of HUVECs as compared to non-targeting control with approximately 33% reduction in the relative wound density after 22 h (Fig. 4b, line diagram and bar graph). Based on our observation that overexpression of Protrudin increases angiogenic tube formation, we next investigated the effect of the overexpression on cell migration by the scratch wound assay (Fig. 4c). The results demonstrated an increase in overall migration profile with nearly 30% increase in relative wound density after 24 h as compared to the Mock control (Fig. 4d, line diagram and bar graph).

To confirm the effect of endogenous Protrudin knockdown on cell migration, we performed xCELLigence™ real-time trans-filter cell migration assays. For this, the cells were allowed to migrate towards growth factor-supplemented media across a filter and the migration profile was recorded for 18 h. HUVECs subjected to Protrudin knockdown migrated markedly slower than controls, with nearly 35% reduction in migration after 12 h (Fig. 4e, migration curve and bar graph). These data are consistent with the transcriptome analysis suggesting that Protrudin plays an important role in EC migration.

### Protrudin knockdown inhibits translocation of mTOR-loaded LE/Lys towards cell periphery

**Fig. 5:**
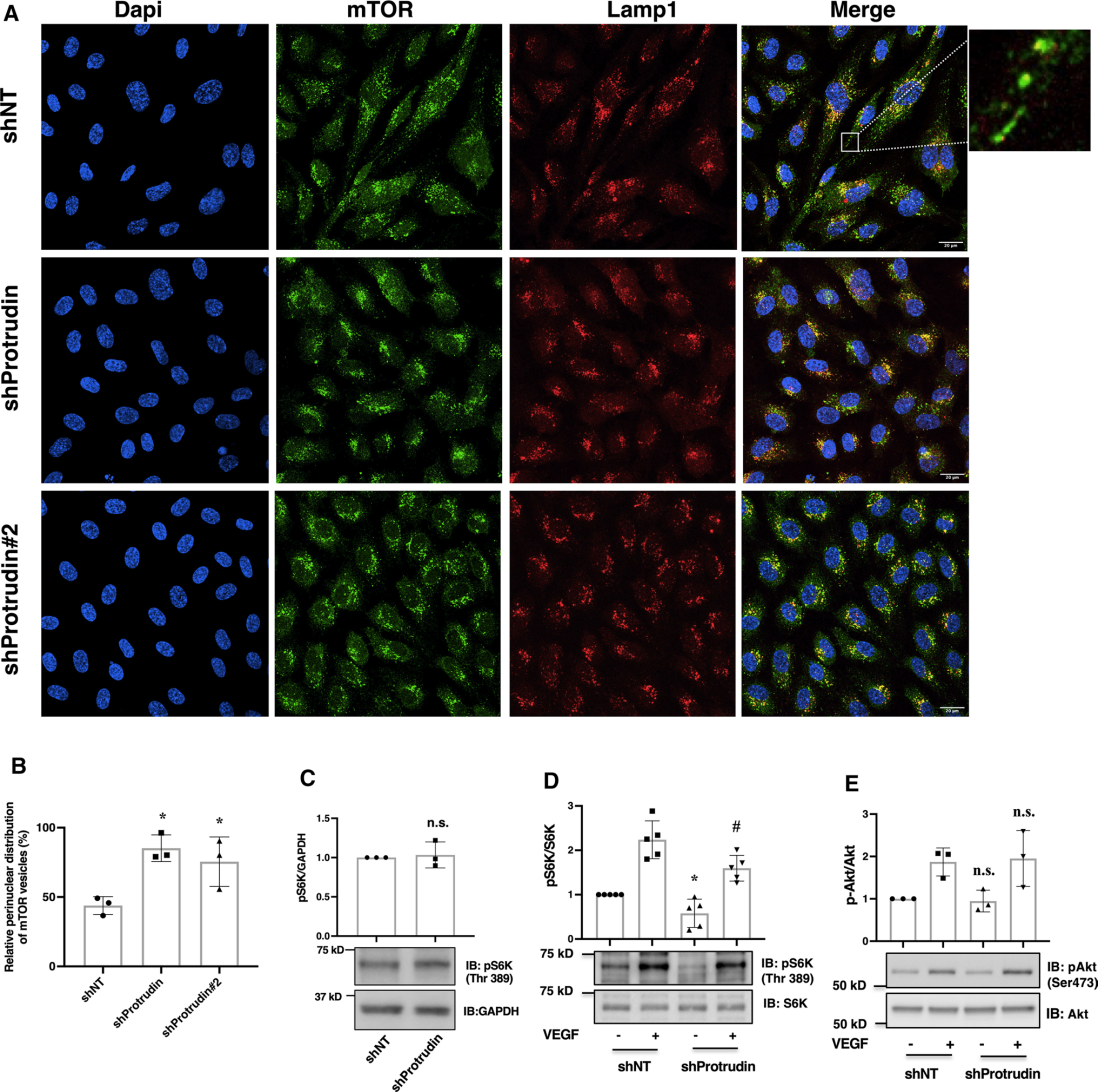
Knockdown of Protrudin promotes perinuclear accumulation of mTOR-positive LE/Lys and diminished mTORC1 activity. **a** Deconvolved confocal micrographs showing distribution of mTOR-positive LE/Lys. HUVECs were seeded on glass coverslips along with lentivirus carrying shProtrudin, shProtrudin#2 or shNT sequence and cells were grown in ECGM2 complete media. Post-transduction, the cells were fixed in 4% PFA and immunostained with anti-Lamp1 and anti-mTOR antibodies. Experiment was done thrice, and the data are representative of at least 15 image captures for each condition per experiment. The high-magnification inset on the right illustrates dispersed mTOR-Lamp1-positive endosomes. **b** Quantification of perinuclear distributed mTOR-positive lysosomes. Graph represents percentage intensities of perinuclearly distributed mTOR relative to total cellular mTOR-positive vesicles. 150 cells per condition were analyzed from 3 independent experiments. Data represent mean ± SD for 3 independent experiments. ^*^p < 0.05 as compared to control samples. **c** Immunoblots showing phosphorylation level of S6K in Protrudin knockdown HUVECs grown in complete media. **d** Immunoblots of Protrudin knockdown or control cells showing the phosphorylation level of S6K. Cells were serum starved in 0.1% BSA for 5 h followed by stimulation with ( +) or without ( −) VEGF (50 ng/ml) for 20 min. Cells were lysed and immunoblotted with total or phospho-p70S6K (Thr 389) antibodies. Data represent mean ± SD for 5 independent experiments.^*^p < 0.05 as compared to shNT, − VEGF samples ^#^*p* < 0.05 compared to shNT, + VEGF samples. **d** Immunoblots showing the phosphorylation level of Akt(Ser473) in Protrudin knockdown HUVECs as described in (**c**). Graphs represent relative densitometric values of pAkt(Ser473) normalized to Akt. Data represent mean ± SD for 3 independent experiments. n.s. indicates statistically not significant compared to shNT, − VEGF samples or shNT, + VEGF samples

Protrudin plays a key role in mTORC1 activation by promoting its PI3P-dependent anterograde translocation in response to growth factors and nutrients [24]. mTORC1 then regulates its downstream effectors 4E-BP1 (eukaryotic initiation factor 4E-binding protein 1) and S6K1 (ribosomal p70 S6 kinase 1). One outcome of this activation is enhanced expression of small GTPases (RhoA, Rac1 and Cdc42) to promote cell migration [9]. We, therefore, investigated if the inhibition of EC migration upon Protrudin knockdown could involve perturbation of mTOR distribution. For this, shProtrudin, shProtrudin#2 (knockdown verified in Suppl. Figure 1b) and shNT HUVECs were stained with antibodies against mTOR and Lamp1. Knockdown of Protrudin resulted in accumulation of mTOR-positive LE/Lys in the perinuclear region as compared to dispersed mTOR vesicles in shNT cells (Fig. 5a, b). However, the inhibition of mTOR translocation did not affect downstream activation of S6 kinase (S6K) in HUVECs grown in nutrient rich conditions (Fig. 5c). We next examined if Protrudin knockdown affects S6K activation in low nutrient/serum-depleted condition and the effect of VEGF stimulation under this condition. Protrudin knockdown cells were serum-starved in ECM2 media (devoid of serum and growth factors but containing amino acids). As shown in Fig. 5d, knockdown of Protrudin resulted here in reduced phosphorylation and activation of S6K both in basal and VEGF-stimulated conditions (lane 1 vs. lane 3 and lane 2 vs. lane 4). However, the phosphorylation of Akt was not affected under these conditions (Fig. 5e, lane 1 vs. lane 3 and lane 2 vs. lane 4). This data suggests that knockdown of Protrudin inhibits basal and VEFG stimulated mTORC1/S6K signaling in endothelial cells only under serum-depleted conditions.

### Protrudin knockdown impairs activation of focal adhesion kinase

**Fig. 6:**
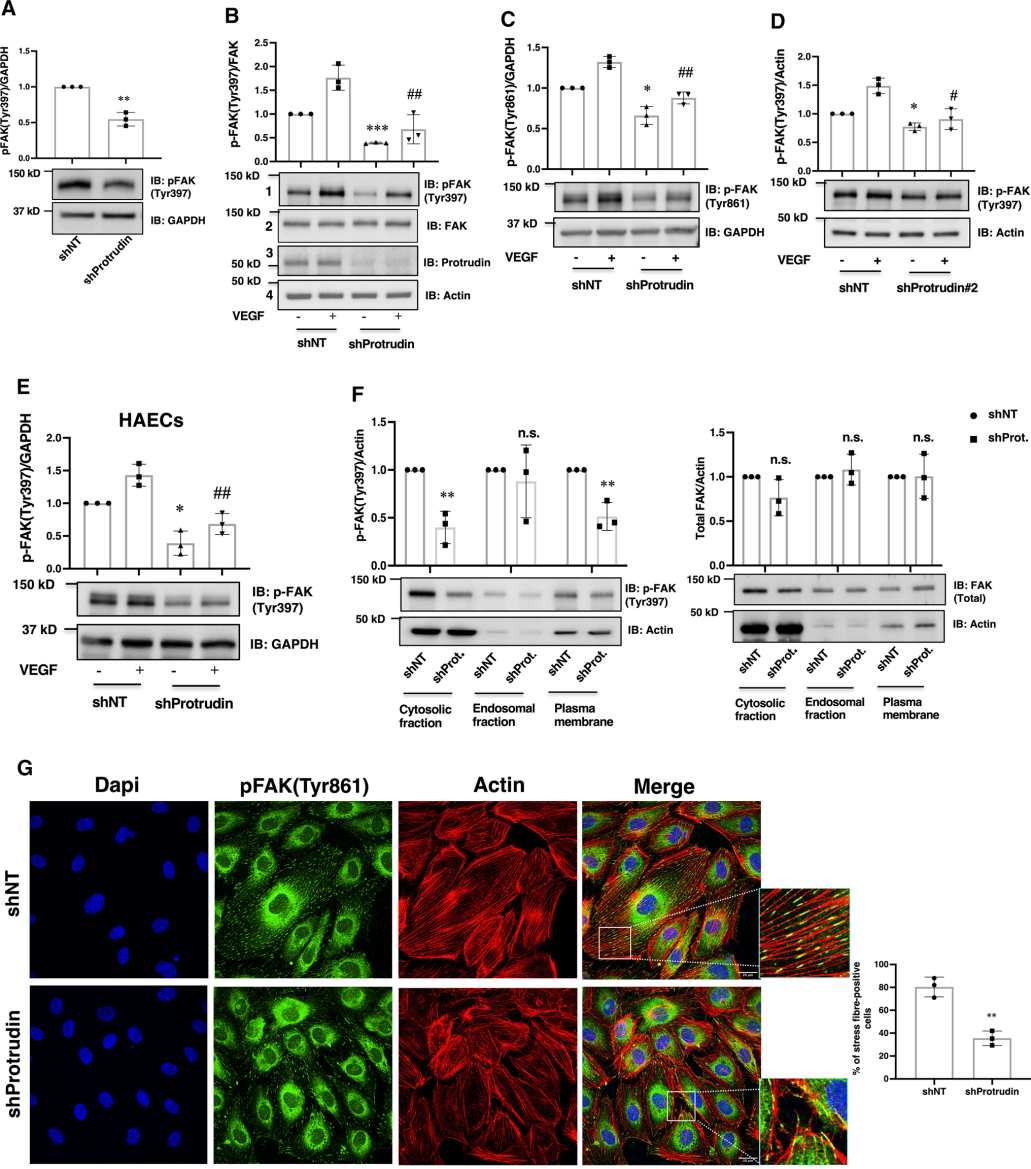
Knockdown of Protrudin impairs activation of focal adhesion kinase. HUVECs were transduced with Protrudin or non-targeting shRNA and incubated for 48 h followed by puromycin selection for 16 h in complete ECGM2 media. **a** Cells were lysed for immunoblotting using antibodies to pFAK(Tyr397) and GAPDH. The experiments were done thrice; a representative blot is shown. The bar diagram represents relative densitometric values of pFAK(Tyr397) normalized for GAPDH. Data represent mean ± SD for three independent experiments, ^**^p < 0.01, compared to shNT. **b** Post-selection shNT or shProtrudin cells were serum-starved for 5 h in ECM2 media containing 0.1%BSA followed by stimulation without ( −) or with ( +) VEGF (50 ng/ml) for 5 min, and lysed for immunoblotting using antibodies to pFAK(Tyr397) (panel 1), total FAK (panel 2); Protrudin (panel 3), actin (panel 4); **c** pFAK(Tyr861) (upper panel), GAPDH (lower panel). The experiments were done thrice; representative blots are shown. The bar diagrams in (**b**, **c**) represent relative densitometric values of pFAK(Tyr397) or pFAK(Tyr861) normalized for total FAK and GAPDH, respectively. Data represent mean ± SD for three independent experiments, ^***^p < 0.001, ^*^p < 0.05 compared to shNT without VEGF stimulation. ^##^*p* < 0.01 compared to shNT with VEGF stimulation. **d** HUVECs were transduced with shProtrudin#2 or non-targeting shRNA as described above for (**a**) and (**b**). Cells were lysed and immunoblotted with anti-pFAK(Tyr 397) (upper panel) or -actin (lower panel). The experiments were done thrice; a representative blot is shown. The bar diagram represents relative densitometric values of pFAK (Tyr397) normalized to actin. Data represent mean ± SD, ^*^p < 0.05 compared to shNT without VEGF stimulation. ^#^*p* < 0.05 compared to shNT with VEGF stimulation. **e** Human aortic endothelial cells (HAECs) were transduced with shProtrudin or non-targeting shRNA as described above. Cells were lysed and immunoblotted using antibodies against pFAK(Tyr 397) (upper panel) and GAPDH (lower panel). The experiments were done thrice; a representative blot is shown. The bar diagrams represent relative densitometric values of pFAK normalized to GAPDH. Data represent mean ± SD, ^*^p < 0.05 compared to shNT without VEGF stimulation. ^##^*p* < 0.01 compared to shNT with VEGF stimulation. **f** Immunoblot showing cytoplasmic, endosomal and plasma membrane fractions of HUVEC transduced with shNT or shProtrudin, serum-starved for 5 h in medium with 0.1% BSA prior to subcellular fractionation as described in *Methods.* Data represent mean ± SD for three independent experiments, ^**^p < 0.01, n.s. indicates statistically not significant. The bar diagrams represent relative densitometric values of pFAK or FAK normalized to actin. **g** HUVECs were seeded on glass coverslips along with control or shProtrudin lentivirus for 48 h in ECGM2 complete media, followed by puromycin selection. Cells were then fixed and stained with Phalloidin-AlexaFluor 594 (actin) or anti-pFAK(Tyr861) and analyzed by confocal microscopy, followed by deconvolution. The experiment was done thrice, and the data are representative of at least 15 image captures per experiment. Bar diagram demonstrates quantification of the percentage of cells with actin stress fibers. 60 cells per condition were analyzed for the quantification, *n* = 3 independent experiments. Data represent mean ± SD. ^**^p < 0.01 compared to shNT, two-tailed Student’s *t*-test

The mTORC1-activated S6K activates the focal adhesion proteins FAK and paxillin to promote cell migration [8]. Focal adhesion (FA) dynamics involving synergy between FA and cytoskeletal proteins is crucial for EC protrusion formation and migration [48]. Therefore, we next studied if downregulation of Protrudin affects FAK activation in ECs. For this, Protrudin knockdown HUVECs grown in complete growth media were lysed to determine the activation status of FAK. Knockdown of Protrudin significantly diminished FAK phosphorylation (at Tyr397) as compared shNT control samples (Fig. 6a). As VEGF signaling is important for endothelial cell migration, we next determined if Protrudin knockdown specifically affects VEGF-mediated FAK activation. For this, cells subjected to Protrudin knockdown were serum-starved for 5 h in media containing 0.1% BSA and then stimulated with or without 50 ng/ml VEGF, followed by western blot analysis. Quantification of the blots revealed a significant reduction of basal as well as VEGF-stimulated FAK phosphorylation at Tyr397 (Fig. 6b, lane 1 vs. lane 3 and lane 2 vs. lane 4) and to a lesser extent at Tyr861 (Fig. 6c, lane 1 vs. lane 3 and lane 2 vs. lane 4). To confirm that the effect of Protrudin knockdown was specific, HUVECs were transduced with a lentivirus carrying another independent shRNA sequence (shProtrudin#2) and the FAK phosphorylation was quantified, revealing a similar decrease in basal and VEGF-stimulated FAK phosphorylation upon Protrudin knockdown (Fig. 6d). To study if this function of Protrudin can also be observed in another primary endothelial cell type, we performed Protrudin knockdown in Human aortic endothelial cells (HAECs). The results demonstrated that, similar to HUVECs, knockdown of Protrudin reduced FAK activation also in these aorta-derived ECs (Fig. 6e). A previous study by Pedersen et al. [24] demonstrated that deletion of Protrudin in MDA-MB-231 breast cancer cells inhibits the invasion of these cells. To confirm our findings on the role of Protrudin in FAK activation, Protrudin knockout MDA-MB-231 cells were stimulated with or without HGF. Immunoblot analysis demonstrated a reduction of FAK activation upon Protrudin deletion in basal as well as HGF stimulated MDA-MB-231 cells (Suppl. Fig. S3).

FAK is recruited onto the endosomes and is activated for cell migration [35]. In this context Protrudin plays an important role in endosome maturation and anterograde endosomal trafficking [17, 49]. Therefore, we next investigated if knockdown of Protrudin affects FAK binding or activation on endosomes. For this, cytoplasmic, endosomal and plasma membrane fractions were collected from shNT and shProtrudin HUVECs. Validation of the fractionated samples in shown as Suppl. Fig. S4. Western immunoblot results showed that FAK phosphorylation was significantly reduced in the cytosolic or plasma membrane fractions of shProtrudin HUVECs, corroborating the notion that Protrudin regulates FAK activity. However, no significant difference in FAK expression and phosphorylation in endosomal fractions was observed, suggesting that inhibition of Protrudin does not affect FAK binding or its activation on endosomes (Fig. 6f). Importantly, confocal microscopy analyses showed a reduction in actin stress fibers with an altered distribution of active FAK in shProtrudin HUVECs compared to shNT cells (Fig. 6g, Image and inset). Migrating endothelial cells possess actin stress fibers with focal adhesion (FA) proteins like FAK anchored at either both or one end of these stress fibers, as seen in our study in shNT control samples (Fig. 6g). These FA proteins are well-aligned as parallel bundles along the longitudinal axis of the cells [50]. However, inhibition of cell migration, as seen in our study upon Protrudin knockdown, is accompanied by loss of stress fibers and the parallel alignment of focal adhesion proteins. These results confirmed that Protrudin indeed regulates FAK activation and suggested that the effects of Protrudin on EC migration are likely due to altered focal adhesion dynamics.

**Fig. 7:**
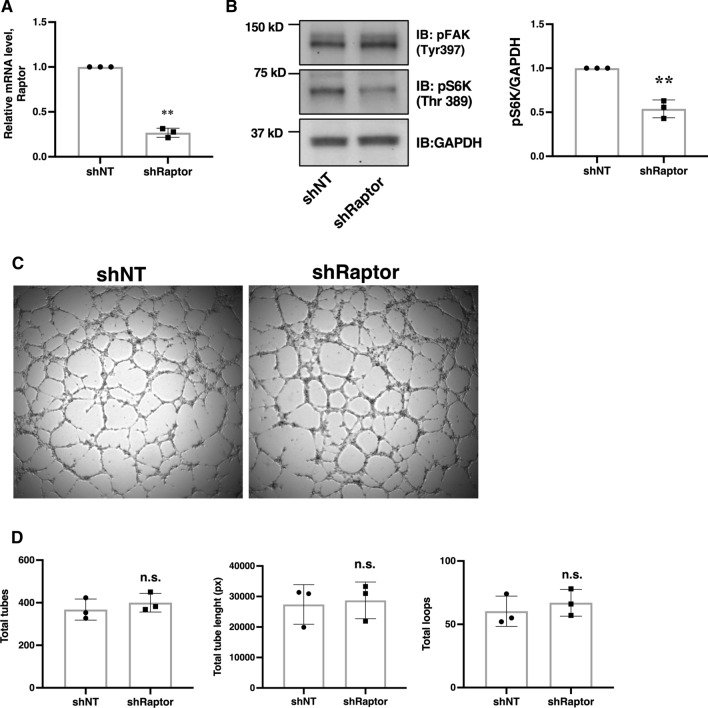
Knockdown of Raptor does not affect FAK activation or angiogenic tube formation in HUVECs. **a** Cells were transduced with lentivirus targeting Raptor or non-targeting control lentivirus (shNT) and incubated in complete ECGM2 growth media for 48 h. Transduced cells were selected with puromycin and lysed for Quantitative RT-PCR. **b** Post-selection Raptor knockdown and shNT cells were subjected to western immunoblotting and probed with anti-pFAK(Tyr397), anti-pS6K(Thr389) and anti-GAPDH antibody. **c** HUVECs were transduced with shNT or shRaptor lentivirus and then seeded on a Millipore matrix as described in Fig. 1a. **d** Bar diagram demonstrating quantification of the number of tubes and loops, as well as the length of tubes in shNT versus shRaptor cells. Data represent mean ± SD of three independent experiments, two-tailed Student’s *t*-test, n.s., statistically not significant

To examine if downregulation of S6K affects FAK activation or angiogenesis, HUVECs were transduced with shRNA lentivirus targeting Raptor (shRaptor). As shown in Fig. 7a, knockdown of Raptor reduced the mRNA level by approximately 75%. This significantly reduced S6K phosphorylation at Thr389. However, the knockdown of Raptor did not affect FAK phosphorylation (Fig. 7b). In addition, knockdown of Raptor did not affect angiogenic tube formation (Fig. 7c, d). These data suggest that the reduction in mTORC1 activity in HUVECs does not significantly affect FAK activation and angiogenesis.

### Vascular progression is retarded in Protrudin/Zfyve27 knockout mice in vivo

**Fig. 8:**
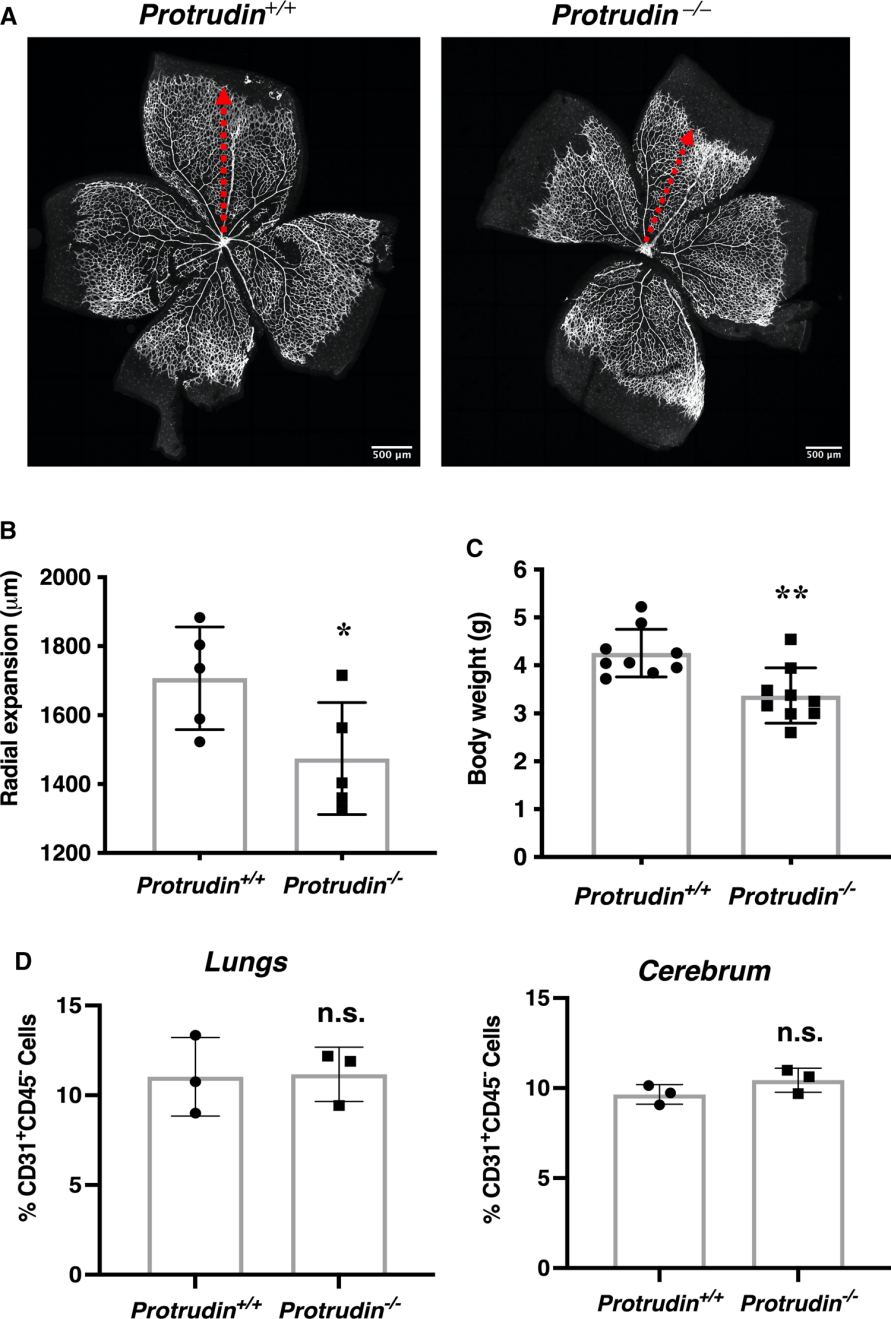
Protrudin gene deletion retards physiological postnatal angiogenesis. **a** Isolectin B4 staining of postnatal day 7 retinas from Zfyve27^+*/*+^ (wild-type) and Protrudin KO (Zfyve27^−/−^) mice, showing vascular progression. Scale bar = 500 μm; *n* = 5. **b** Bar graph representing quantification of retinal vascular progression; *n* = 5. Data represent mean ± SD, *p < 0.05, two-tailed Student’s *t*-test. **c** Body weight of Zfyve27^+*/*+^ and Zfyve27^*–/–*^ mice at 7 to 9 days of age, n = 9, two-tailed Student’s t-test. Data represent mean ± SD, **p < 0.01. **d** FACS-based cell counts for CD31^+^CD45^—^ cell populations isolated for mice lungs (postnatal day 7, *n* = 3) and cerebrum (postnatal day 13–16, *n* = 3). n.s., statistically not significant

We next explored the role of Protrudin in angiogenesis in vivo*.* For this, we analyzed the development of retinal vasculature in postnatal day 7 mice. Mice with global knockout of Protrudin (also known as Zfyve27) demonstrated a significant retardation of retinal vascular progression as compared to the wild-type littermates (Fig. 8a, b). As described earlier for adult mice (31), the postnatal day 7 mice displayed significantly lower body weight than the wild-type littermates (Fig. 8c). Interestingly, there was no difference in the percentage of CD31^+^CD45^−^ EC population isolated from the lungs and cerebrum of these mice, ruling out the possibility of a defect in growth or survival of ECs in the Protrudin knockout mice (Fig. 8d). The observed retardation of vascular progression thus supports a function of Protrudin in angiogenesis in vivo. However, generation and analysis of an EC-specific knockout will be necessary to firmly demonstrate the role of Protrudin in vascular development.

## Discussion

Cytoskeletal reorganization and cell migration are primary events in the process of angiogenesis. This has relevance to physiological functions such as wound healing and pathological conditions such as cancer and cardiovascular diseases. In the present study we unravel a novel role of an ER-endosome membrane contact site protein, Protrudin, in endothelial cell (EC) biology and angiogenesis per se. Our major findings establish that Protrudin is necessary for angiogenic tube formation by primary endothelial cells, HUVECs, in vitro and suggest retardation of retinal vascular progression in vivo in Protrudin/Zfyve27^−/−^ mice.

A study by Raiborg et al. [17] demonstrated the role of Protrudin in anterograde transport and plasma membrane fusion of endosomes required for neurite outgrowth, mediated by kinesin loading on the endosomes. While overexpression of wild-type Protrudin stimulated angiogenic tube formation by HUVECs, constructs with kinesin (ΔKIF) or endosome (ΔFYVE ΔLCR) binding domains deleted showed an inhibitory effect on the angiogenic tube formation, suggesting that these interactions are crucial determinants of Protrudin function in angiogenesis in vitro. This is consistent with the notion that endosome transport is an important aspect of Protrudin activity in EC that promotes endothelial cell protrusion formation and migration. This is also supported by transcriptome analysis in the present study identifying cell migration as a major cellular function altered upon Protrudin knockdown in HUVECs. Of note, gene expression of Rac1 and MAPK3 that play key roles in VEGF-mediated endothelial cell migration [51–53] was significantly downregulated upon Protrudin knockdown.

Expression of small GTPases (Rac1, RhoA and Cdc42) is regulated by the mTORC1 effectors S6K and 4E-BP1 [9]. Rapamycin sensitive mTORC1/S6K signaling is a master regulator of cell growth and migration, and inhibition of this signaling pathway has significant therapeutic potential to inhibit tumor angiogenesis [54, 55]. The mTORC1-activated S6K1 pathway plays an important role in VEGF-A mediated angiogenesis, wherein overexpression of S6K1 in a mice model enhances VEGF-A mediated angiogenesis [56]*.* However, a comparative study addressing the contribution of mTORC1 and mTORC2 to vascular assembly highlighted the importance of mTORC2 in angiogenesis. In endothelial cells isolated from Raptor-knockout mice, there was only a mild effect on VEGF-induced endothelial cells function [57]. This is in line with our findings that knockdown of Raptor does not affect angiogenic tube formation. A previous study by Hong et al. [25] demonstrated that the Protrudin-mediated transport of LE/Lys promotes peripheral distribution of these organelles and thus the activity of mTOR in different cell lines including HeLa, HEK293 and RPE1 cells. Inhibition of Protrudin accumulates mTOR-positive LE/Lys near the perinuclear region and inhibits their dispersal towards cell periphery in the presence of nutrients and growth factors, which in turn inhibits S6K activation [25]. Our findings indicate that although knockdown of Protrudin precludes peripheral distribution mTOR vesicles, however, it affects VEGF-stimulated S6K activation under low nutrient conditions only. Therefore, it remains unclear how mTOR clustering upon Protrudin knockdown could affect endothelial cell functions.

Focal adhesion dynamics and FAK activation in particular play a key role in endothelial cell protrusion formation. Polarized FAK distribution is a key determinant of directional cell migration [48]. Several studies have demonstrated a crucial role of FAK in angiogenesis and have proposed FAK as a potential target for anti-angiogenesis-based therapeutics [58–60].

Our results demonstrate that Protrudin regulates FAK activation and thereby its distribution in primary endothelial cells. Furthermore, FAK is reported to be recruited onto endosomes, this localization being required for FAK activation and subsequently cell migration [35, 61]. Our findings suggest that depletion of Protrudin does not directly affect the binding or activation of FAK on endosomes. Therefore, it is likely that Protrudin affects FAK activity by an indirect mechanism. Our findings rule out the possibility the FAK activation could be affected by inhibition of mTORC1/S6K signaling axis as knockdown of Raptor did not affect FAK activation. Therefore, further studies are required to understand how Protrudin downregulation affects FAK activation in endothelial cells. Experimental screening to identify the different cargo molecules that are anterogradely transported by Protrudin in endothelial cells could be helpful in addressing this question. Nevertheless, our study is the first to show that Protrudin does regulate endothelial cell functions like migration and angiogenic tube formation, and FAK plays a key role in this function.

Protrudin plays an important role in cancer cell invasiveness, and overexpression of Protrudin in non-cancerous RPE1 cells promoted invadopodia formation in these cells. Furthermore, a high Protrudin expression level was found to be correlated with a lower survival probability of ovarian, gastric and breast cancer patients [24]. In the present study, we show that overexpression of Protrudin is associated with increased angiogenic tube formation and migration capacity of endothelial cells in vitro. In the future, it will be intriguing to study the role of Protrudin in tumor angiogenesis and explore its therapeutic potential against tumor growth and metastasis.

In vivo deletion of Protrudin does not affect survival in mice. However, postnatally, the mice have a lower body weight and are smaller in size, an observation reported previously also for the adult animals [26]. This is associated with a slower vascular progression as observed here during the development of retinal vasculature. Consistent with the reduced body size, we observed that the total number of cells isolated from mice cerebrum and lungs was lower in the Protrudin/Zfyve27^−/−^ animals than the wild-type littermates; However, the percentage of CD31^+^CD45^−^ endothelial cells remained unaltered between the knockout and wild-type littermates, indicating the absence of a defect in growth and survival of endothelial cells in these tissues. Thus, although our work revealed a significant retardation in the progression of retinal vasculature, possibly due to an endothelial cell migration defect in the Protrudin knockouts, the global deletion of this protein might cause confounding effects that can only be eliminated by generating an EC-specific knockout model.

To conclude, this study is the first to report the role of Protrudin in endothelial cell function and angiogenesis. Our findings suggest that the protein executes important functions in endothelial cells, with significant impacts on FAK signaling, cell migration and angiogenic capacity. Future study employing an EC-specific knockout mouse model is required to pinpoint in detail the molecular functions of Protrudin in endothelia in vivo.

### Supplementary Information

Below is the link to the electronic supplementary material.

Supplementary file1 (DOCX 4435 KB)

Supplementary file2 (XLSX 3830 KB)

## Data Availability

The datasets generated during and/or analyzed during the current study are available from the corresponding author on reasonable request.

## Declarations

### Animal welfare

All animal experiments and procedures were approved by the Regional Administrative Agency of Southern Finland under the license ESAVI/16352/2019.
