# Supplementary material for: Protrudin regulates FAK activation, endothelial cell migration and angiogenesis
Source: Cell Mol Life Sci. 2022 Apr 4;79(4):220. doi: 10.1007/s00018-022-04251-z (PMC8977271; doi:10.1007/s00018-022-04251-z)

**Protrudin regulates FAK activation, endothelial cell migration and angiogenesis**

Amita Arora^1^, Annukka M Kivelä^1^, Ling Wang^2,3^, Rimante Minkeviciene^1^, Juuso H Taskinen^1^, Birong Zhang^4,5^, Annika Koponen^1^, Jing Sun^4,5^, Michiko Shirane^6^, You Zhou^4,5^, Pirta Hotulainen^1^, Camilla Raiborg^2,3^, Vesa M Olkkonen^1,7^*

^1^Minerva Foundation Institute for Medical Research, Helsinki, Finland

^2^Centre for Cancer Cell Reprogramming, Faculty of Medicine, University of Oslo, Oslo, Norway

^3^Department of Molecular Cell Biology, Institute for Cancer Research, Oslo University Hospital, Oslo, Norway

^4^Systems Immunity Research Institute, Cardiff University School of Medicine, Cardiff University, Cardiff, United Kingdom.

^5^Division of Infection and Immunity, Cardiff University School of Medicine, Cardiff University, Cardiff, United Kingdom

^6^Department of Molecular Biology, Graduate School of Pharmaceutical Sciences, Nagoya City University, Nagoya, Aichi, Japan

^7^Department of Anatomy, Faculty of Medicine, University of Helsinki, Helsinki, Finland

**Key words:** endosomes, focal adhesion kinase, mTOR, vasculature, Zfyve27

**Running title:** Protrudin function in endothelial cells

*Author for correspondence: Vesa Olkkonen, Minerva Foundation Institute for Medical Research, Biomedicum 2U, Tukholmankatu 8, FI-00290 Helsinki, Finland.

Tel. +358-50-4112297, E-mail: vesa.olkkonen@helsinki.fi

**Supplementary Figure legends**

**Fig. S1** Western blot to determine the efficiency of Protrudin knockdown/overexpression in lentivirally transduced HUVECs. **(A and B)** shProtrudin **(A)**, shProtrudin#2 **(B)** or shNT transduced cells were subjected to puromycin selection for 16 h. Cells were then lysed and probed with anti-Protrudin (top) and -GAPDH (bottom). Number of experiments= 4 for **(A)** and =3 for **(B)**; a representative blot is shown. The bar diagram represents relative densitometric values of Protrudin after normalizing for GAPDH expression. Data represents mean ± SD, ***p<*0.01, ****p<*0.001. **(C)** Western blot validating the overexpression of shRNA-resistant Protrudin (ProtrudinWtRescue) upon Protrudin knockdown **(D)** Western blot showing the expression of Protrudin upon lentiviral transduction of wild-type or domain deleted Protrudin constructs. Blots of total cell protein were probed with anti-Protrudin (top) or anti-GAPDH (bottom).

**Fig. S2** Modulation of Protrudin expression does not induce ER stress and cell death in HUVECs. **(A)** shProtrudin or shNT knockdown cells were lysed and western immunoblotted with anti-phospho-PERK, anti-phospho-eIF2α or anti-tubulin antibody. HUVECs treated with DMSO or thapsigargin (10 μM, for 3 h) were used as positive control for ER stress. **(B)** Post-puromycin selection, shProtrudin or shNT cells were fixed in 1% PFA and stained for TUNEL apoptosis assay. **(C)** Post-selection cells were subjected to RealTime-Glo^TM^ Annexin V Apoptosis and Necrosis Assay. **(D)** Cells overexpressing Protrudin (Wild-type or mutant) lentiviral constructs were subjected to RealTime-Glo^TM^ Annexin V Apoptosis and Necrosis Assay.

**Fig. S3** Protrudin depleted MDA-MB-231 cells display reduced levels of FAK-Tyr397 phosphorylation. Western blot and graph representing the relative FAK-Tyr397 phosphorylation between parental (P) or protrudin knock out (KO) MDA-MB-231 cells treated or not with 50 ng/ml HGF for 30 min. Error bars denote ± SEM from 4 independent experiments indicated by different colours. All conditions vs. parental -HGF set to 1: One-sample t-test. Parental +HGF vs. KO +HGF: Unpaired t-test.

**Fig. S4** Validation of the fractionated samples. Western blot representing markers for cytoplasmic (Tubulin), endosomal (EEA1) and plasma membrane (Orai1) fractions. Experiment was done thrice and a representative image is shown.

**Fig. S1**


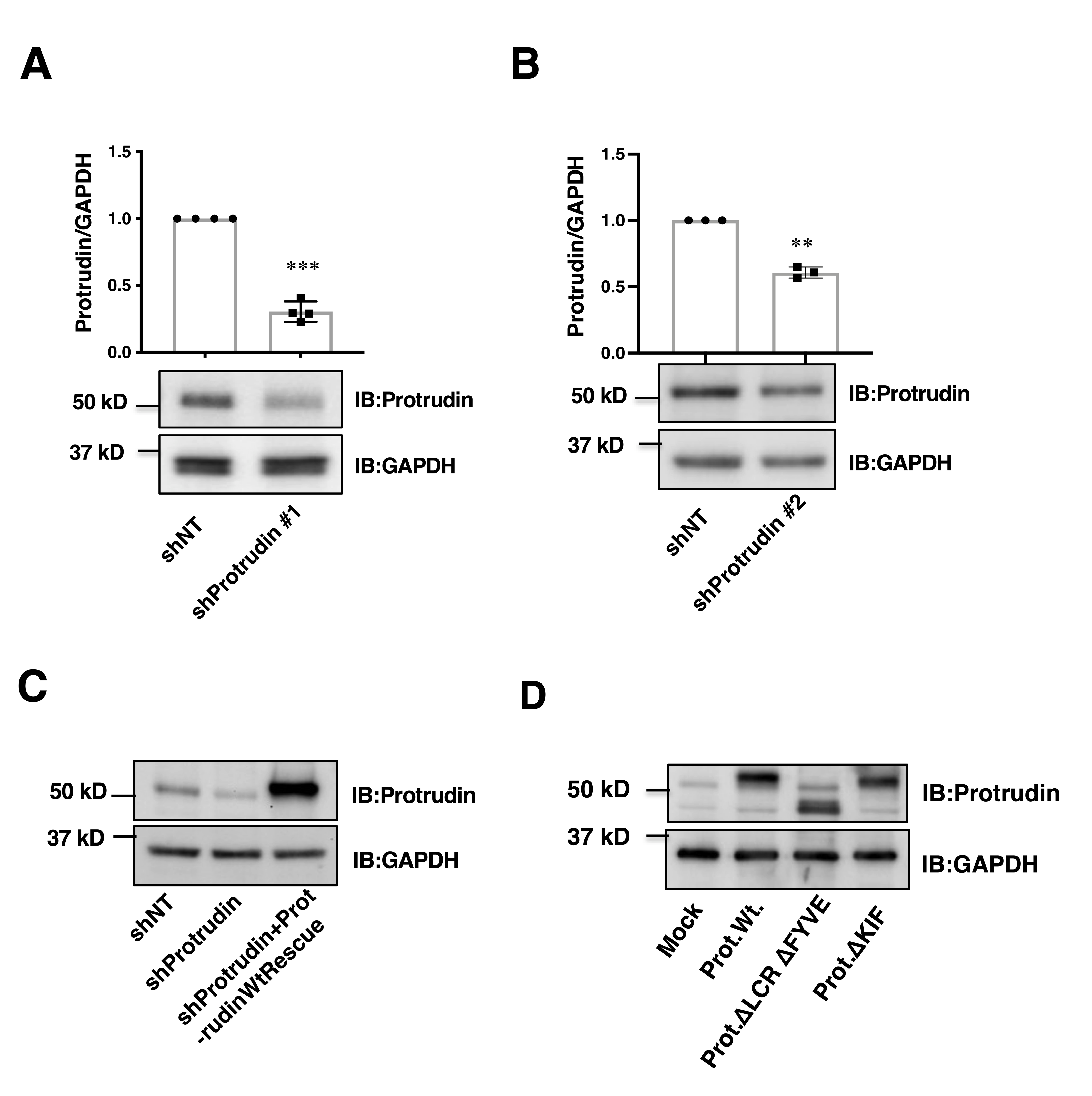


**Fig. S2**


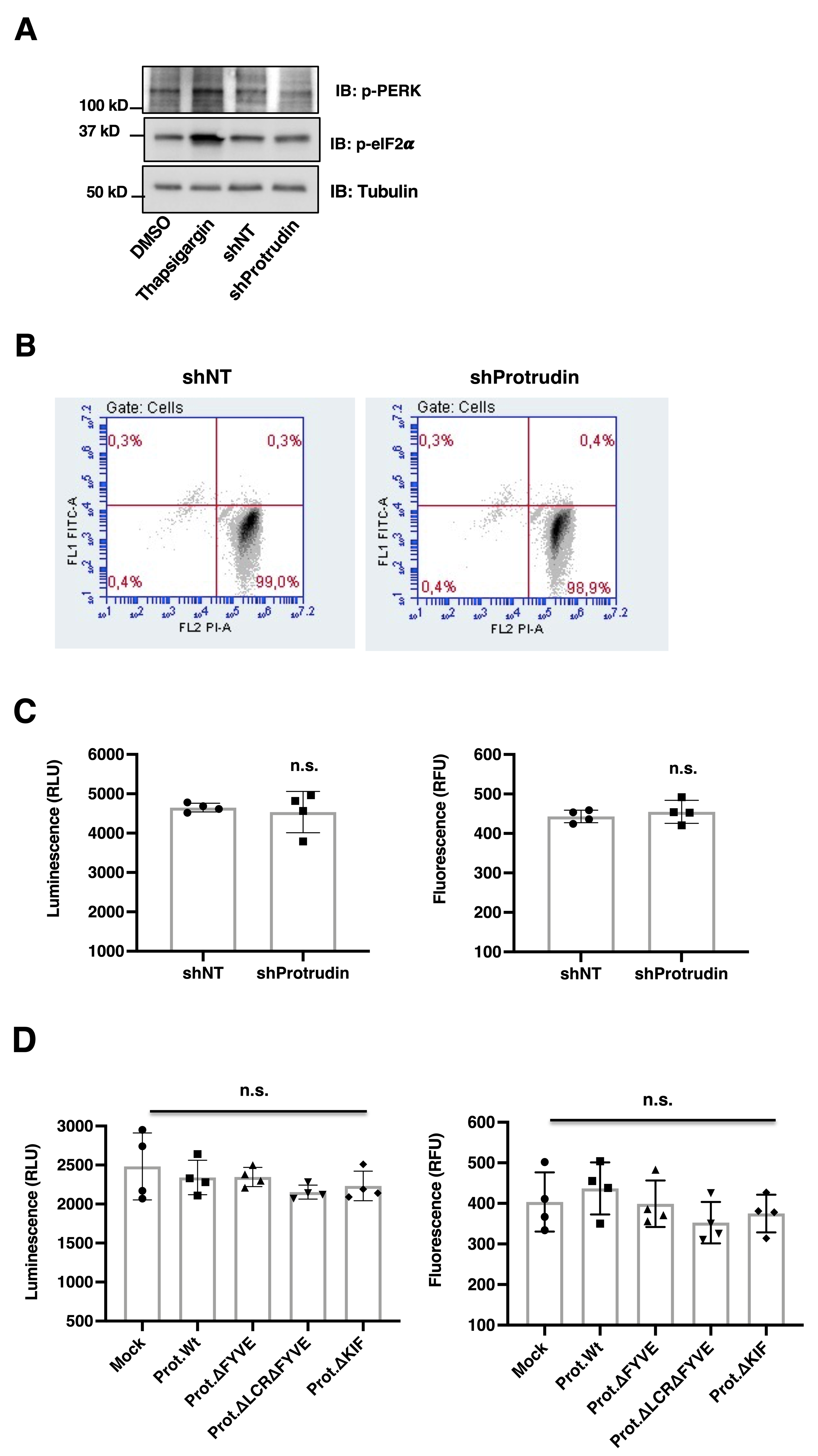


**Fig. S3**

**Fig. S4**


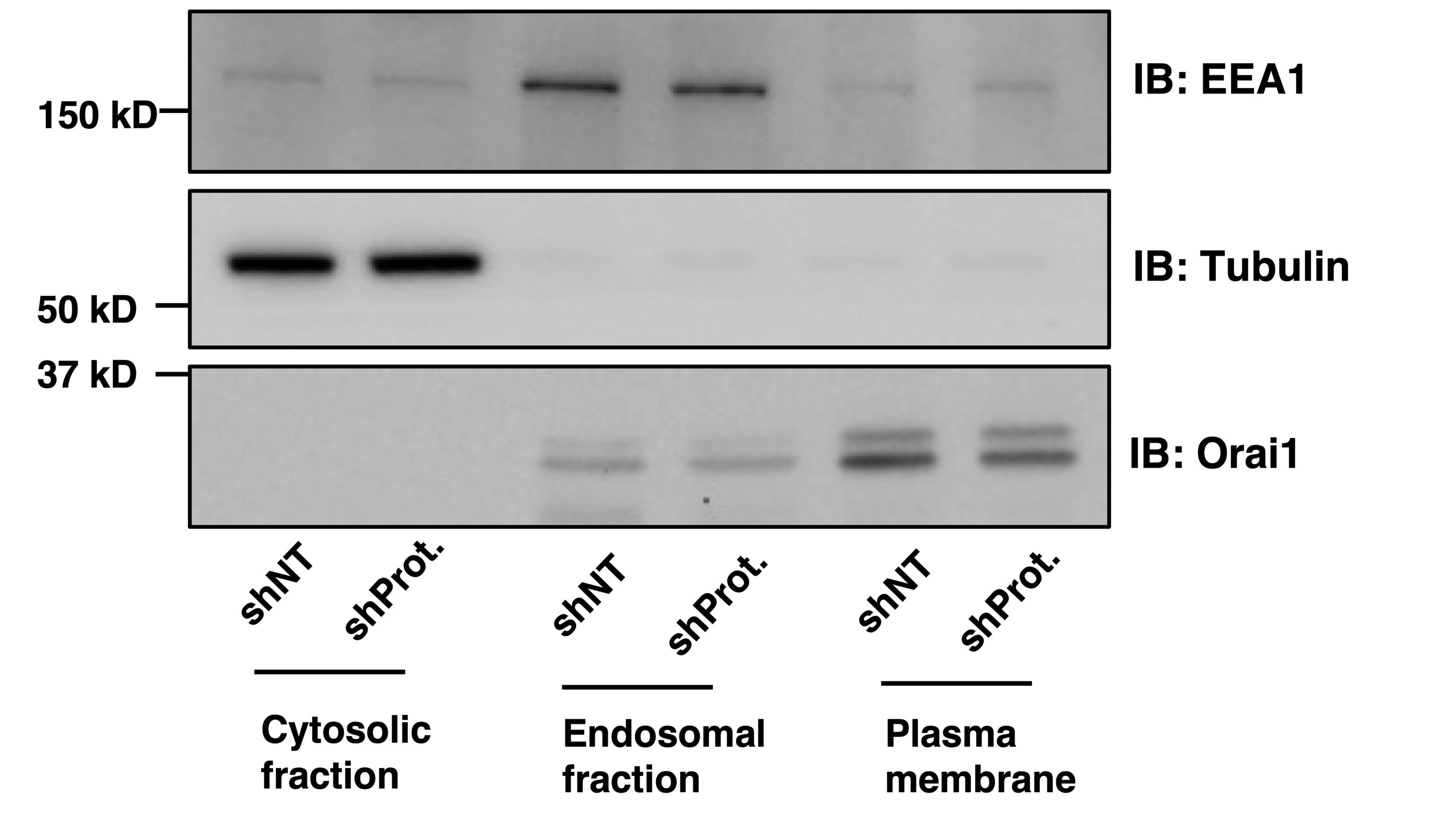

Supplement: Supplementary file 1 — Supplementary file1 (DOCX 4435 KB) [file 18_2022_4251_MOESM1_ESM.docx]
